# Demographics, Outcomes, and Risk Factors for Patients with Sarcoma and COVID-19: A CCC19-Registry Based Retrospective Cohort Study

**DOI:** 10.3390/cancers14174334

**Published:** 2022-09-05

**Authors:** Michael J. Wagner, Cassandra Hennessy, Alicia Beeghly, Benjamin French, Dimpy P. Shah, Sarah Croessmann, Diana Vilar-Compte, Erika Ruiz-Garcia, Matthew Ingham, Gary K. Schwartz, Corrie A. Painter, Rashmi Chugh, Leslie Fecher, Cathleen Park, Olga Zamulko, Jonathan C. Trent, Vivek Subbiah, Ali Raza Khaki, Lisa Tachiki, Elizabeth S. Nakasone, Elizabeth T. Loggers, Chris Labaki, Renee Maria Saliby, Rana R. McKay, Archana Ajmera, Elizabeth A. Griffiths, Igor Puzanov, William D. Tap, Clara Hwang, Sheela Tejwani, Sachin R. Jhawar, Brandon Hayes-Lattin, Elizabeth Wulff-Burchfield, Anup Kasi, Daniel Y. Reuben, Gayathri Nagaraj, Monika Joshi, Hyma Polimera, Amit A. Kulkarni, Khashayar Esfahani, Daniel H. Kwon, Luca Paoluzzi, Mehmet A. Bilen, Eric B. Durbin, Petros Grivas, Jeremy L. Warner, Elizabeth J. Davis

**Affiliations:** 1 Department of Medicine, University of Washington, and Clinical Research Division, Fred Hutchinson Cancer Center, Seattle, WA 98109, USA; 2 Department of Biostatistics, Vanderbilt University Medical Center, Nashville, TN 37232, USA; 3 Department of Medicine, Division of Epidemiology, Vanderbilt University Medical Center, Nashville, TN 37232, USA; 4 Department of Population Health Sciences, UT Health San Antonio, San Antonio, TX 78229, USA; 5 Department of Medicine, Division of Hematology and Oncology, Vanderbilt University, Nashville, TN 37232, USA; 6 Department of Infectious Diseases, Instituto Nacional de Cancerología, Mexico City 14080, Mexico; 7 Department of Medicine, Columbia University Irving Medical Center, New York, NY 10032, USA; 8 Broad Institute of MIT and Harvard, Cambridge, MA 02142, USA; 9 Department of Hematology and Oncology, University of Michigan, Ann Arbor, MI 48109, USA; 10 University of Cincinnati Cancer Center, Cincinnati, OH 45267, USA; 11 Sylvester Comprehensive Cancer Center, University of Miami, Miami, FL 33136, USA; 12 Department of Investigational Cancer Therapeutics, University of Texas MD Anderson Cancer Center, Houston, TX 77030, USA; 13 Department of Medicine, Stanford University, Stanford, CA 94304, USA; 14 Department of Medical Oncology, Dana-Farber Cancer Institute, Boston, MA 02215, USA; 15 Department of Medicine, Moores Cancer Center, University of California San Diego, San Diego, CA 92037, USA; 16 Department of Medicine, Roswell Park Comprehensive Cancer Center, University of Buffalo, Buffalo, NY 14203, USA; 17 Department of Medical Oncology, Memorial Sloan-Kettering Cancer Center, New York, NY 10065, USA; 18 Henry Ford Cancer Institute, Detroit, MI 48202, USA; 19 Department of Radiation Oncology, The Ohio State University Comprehensive Cancer Center, Columbus, OH 43210, USA; 20 Division of Hematology and Medical Oncology, Knight Cancer Institute at Oregon Health and Science University, Portland, OR 97239, USA; 21 Department of Medicine, The University of Kansas Medical Center, Kansas City, KS 66103, USA; 22 Hollings Cancer Center, Medical University of South Carolina, Charleston, SC 29425, USA; 23 Division of Hematology and Oncology, Loma Linda University School of Medicine, California, CA 92354, USA; 24 Department of Medicine, Division of Hematology-Oncology, Penn State Cancer Institute, Hershey, PA 17033, USA; 25 Masonic Cancer Center, University of Minnesota, Minneapolis, MN 55455, USA; 26 Segal Cancer Centre, Jewish General Hospital, McGill University, Montreal, QC H4A 3J1, Canada; 27 UCSF Helen Diller Family Comprehensive Cancer Center, University of California San Francisco, California, CA 94117, USA; 28 Department of Oncology, Albert Einstein College of Medicine, Montefiore Medical Center, Bronx, NY 10467, USA; 29 Department of Oncology, Emory University, Atlanta, GA 30322, USA; 30 Division of Biomedical Informatics, College of Medicine, University of Kentucky, Lexington, KY 40536, USA; 31 Department of Biomedical Informatics, Vanderbilt University, Nashville, TN 37232, USA

**Keywords:** sarcoma, COVID-19, SARS-CoV-2, CCC19

## Abstract

**Simple Summary:**

Sarcomas are a group of cancers with differing clinical features, some of which require long courses of cytotoxic chemotherapy. Therefore, patients with sarcoma may be at high risk of developing severe COVID-19. The aim of our study was to describe risk factors and clinical outcomes for patients with sarcoma and COVID-19. We show that patients with sarcoma have high rates of complications from COVID-19. Risk factors for more severe COVID-19 included older age, poor performance status, and lung metastases. We also compared 30 day mortality rates to a matched cohort of patients with sarcoma without COVID-19 and found that patients with bone sarcoma may be at higher risk of death from COVID-19 than patients with other sarcoma subtypes.

**Abstract:**

Background: Patients with sarcoma often require individualized treatment strategies and are likely to receive aggressive immunosuppressive therapies, which may place them at higher risk for severe COVID-19. We aimed to describe demographics, risk factors, and outcomes for patients with sarcoma and COVID-19. Methods: We performed a retrospective cohort study of patients with sarcoma and COVID-19 reported to the COVID-19 and Cancer Consortium (CCC19) registry (NCT04354701) from 17 March 2020 to 30 September 2021. Demographics, sarcoma histologic type, treatments, and COVID-19 outcomes were analyzed. Results: of 281 patients, 49% (*n* = 139) were hospitalized, 33% (*n* = 93) received supplemental oxygen, 11% (*n* = 31) were admitted to the ICU, and 6% (*n* = 16) received mechanical ventilation. A total of 23 (8%) died within 30 days of COVID-19 diagnosis and 44 (16%) died overall at the time of analysis. When evaluated by sarcoma subtype, patients with bone sarcoma and COVID-19 had a higher mortality rate than patients from a matched SEER cohort (13.5% vs 4.4%). Older age, poor performance status, recent systemic anti-cancer therapy, and lung metastases all contributed to higher COVID-19 severity. Conclusions: Patients with sarcoma have high rates of severe COVID-19 and those with bone sarcoma may have the greatest risk of death.

## 1. Introduction

Since March 2020, the COVID-19 pandemic has significantly impacted hospital systems, patients, and public health in the United States, including disruption of cancer screening, diagnosis, and treatment. This is anticipated to increase both morbidity and mortality of individuals with cancer in upcoming years [1]. In addition to the disruption of care, having a cancer diagnosis is a significant negative prognostic indicator for patients with COVID-19. Patients with active malignancies experience more severe COVID-19 and higher death rates from SARS-CoV-2 infection [2,3,4,5]. In March 2020, the COVID-19 and Cancer Consortium (CCC19) was founded by five institutions with the goal of collecting and disseminating uniformly organized information on patients with cancer and COVID-19. Since then, CCC19 has grown to 125 institutions with >600 collaborators [1]. Analyses of the collective CCC19 patient cohort have demonstrated high 30 day all-cause mortality among patients with both cancer and COVID-19, attributed to both general risk factors including advanced age, male sex, former smoker status, higher number of comorbidities and poor Eastern Cooperative Oncology Group (ECOG) performance status, as well as cancer-specific risk factors such as active (measurable) disease, malignancy type, and recent receipt of systemic chemotherapy [6,7].

Sarcomas are a heterogenous group of mesenchymal tumors that range in severity and risk of recurrence or spread [8]. They include over 170 histologic diagnoses that, depending on the specific subtype and extent of disease, may require chemotherapy or targeted therapy, radiotherapy, surgery, or a combination of treatments [9]. Due to the rarity and diversity of sarcoma subtypes, National Comprehensive Cancer Network (NCCN) guidelines state that all patients with sarcoma should have their case reviewed at a multidisciplinary sarcoma center; this often requires patients to travel long distances which has been shown to significantly impact outcomes of patients with sarcoma [10].

In early 2020 the clinical challenges of sarcomas were further complicated with the emergence of the SARS-CoV-2 virus. Questions arose over which patients might be at increased risk of developing more severe cases of COVID-19, and whether or not it might be safe to travel to sub-specialty sarcoma centers. Due to the need to travel to sarcoma referral centers, patients with sarcoma may be more vulnerable and have higher risk of contracting COVID-19 because they could not isolate at home to the same degree as other patients with cancer. Furthermore, some sarcoma subtypes require intense immunosuppressive chemotherapies that can significantly impact the clinical outcomes of a SARS-CoV-2 infection [11,12,13]. Finally, patients with advanced sarcoma often have metastatic involvement in the lungs, the organs most vulnerable to the consequences of COVID-19. Taken together, patients with both sarcoma and COVID-19 may face more challenges than patients with other cancer types. An early case series suggested that patients with sarcoma have a high risk of developing severe COVID-19 [14]. As the response to the COVID-19 pandemic continues to evolve, an understanding of the unique risk factors faced by patients with sarcoma can help inform decision making for patients and guide advice given by treating physicians. The recent omicron wave of COVID-19 demonstrated that, even two years after the first COVID-19 cases were diagnosed, the possibility of additional variants and additional surges remains. No large study has formally assessed the risks faced by patients with sarcoma and COVID-19; herein, we describe the largest such study to date. 

## 2. Materials and Methods

### 2.1. Study Design and Patient Population

**Figure 1:**
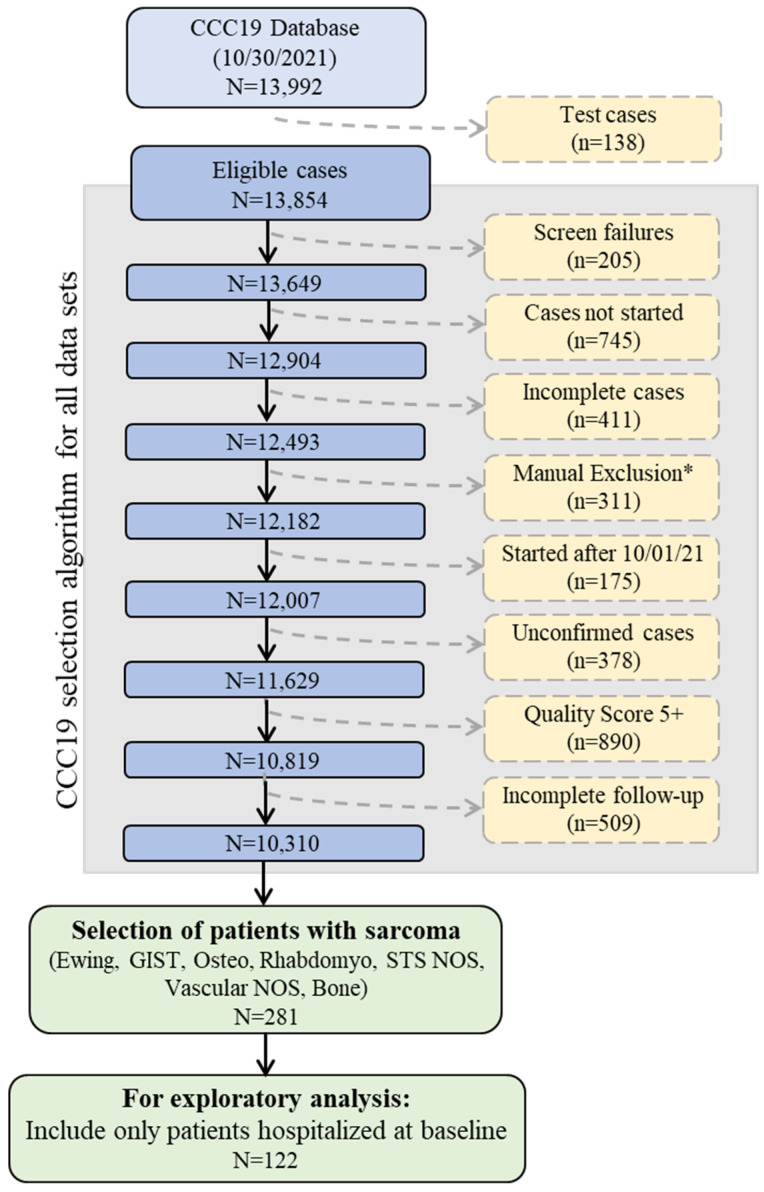
Schematic of CCC19 Database selection. Abbreviations: Gastrointestinal stromal tumor (GIST), Osteosarcoma (Osteo), Rhabdomyosarcoma (Rhabdomyo), Soft tissue sarcoma not otherwise specified (STS NOS). * Manual Exclusions = Duplicate records; In situ solid malignancy; Precursor hematologic condition; Benign hematologic condition; False positive SARS-CoV-2 test; Non-melanoma skin cancer, non-invasive; and Low quality score, non-CCC19 site.

This was a registry-based retrospective cohort study of patients reported to CCC19. Data were collected using a REDCap database housed at the Vanderbilt University Medical Center (VUMC). Details of the CCC19 workflow and data elements have been previously described [1]. Patient reports entered into the CCC19 registry (NCT04354701) between 17 March 2020 and 30 September 2021 were included in the analysis. Supplementary File S1 lists all contributing institutions. Patients with a diagnosis of sarcoma and a laboratory-confirmed SARS-CoV-2 infection were included; those under 18 were truncated to age = 18. Patient reports with inadequate data quality (quality score ≥5 according to our previously published metric) or incomplete primary outcome data were excluded. A diagnosis of sarcoma was further defined as Ewing sarcoma, gastrointestinal stromal tumor (GIST), osteosarcoma, Rhabdomyosarcoma, soft tissue sarcoma not otherwise specified (NOS), vascular sarcoma NOS, or bone sarcoma. Sarcoma subgroups were defined as soft tissue sarcoma (STS), bone sarcoma, GIST, and other/indolent histologies (Figure 1, Supplemental Table S1).

### 2.2. Data Elements

The CCC19 data dictionary can be accessed at https://github.com/covidncancer/CCC19_dictionary. Data collected included baseline demographic data, approximate date of COVID-19 diagnosis, comorbidities, cancer-specific data (sarcoma subtype, extent of disease, metastatic sites), and timing and modality of most recent anti-cancer therapy [7]. Laboratory measurements included absolute lymphocyte count, absolute neutrophil count, white blood cell count, creatinine, lactate dehydrogenase (LDH), and D-dimer. The primary outcome was COVID-19 severity, a six-level ordinal variable ranging from: none of the following events; hospitalization, without or with supplemental oxygen; admission to an intensive care unit (ICU); mechanical ventilation; or death due to any cause. This ordinal severity outcome was meant to capture the full range of clinical complications experienced by patients and was assessed over patients’ total follow-up time. The secondary outcome was 30 day all-cause mortality. 

### 2.3. Statistical Analysis

Standard descriptive statistics were used to summarize baseline patient characteristics and outcomes overall and stratified by sarcoma subtype. Laboratory measurements were summarized among patients hospitalized at baseline. Unadjusted descriptive statistics are presented without formal statistical hypothesis testing (i.e., *p* values). Multiple imputation (twenty iterations; missingness rates were <15% for risk factors other than laboratory measurements) using additive regression, bootstrapping, and predictive mean matching was used to impute missing and unknown data, except unknown ECOG performance status and unknown cancer status, which were included as unknown categories. Imputation was performed on the full dataset before exclusions. Formal statistical hypothesis testing was performed using adjusted odds ratios and 95% confidence intervals for the ordinal COVID-19 severity outcome, which were obtained from ordinal logistic regression models with an offset for (log) follow-up time. Due to the sample size and corresponding degrees of freedom, a reduced set of covariates was included in adjusted models based on a priori clinical relevance [15]. Covariates included age (regression spline with a knot at age 40 years), sex, renal disease, ECOG performance status, site of metastasis (lung vs. none and other vs. none), and time period of COVID-19 diagnosis. Due to substantial collinearity between sarcoma subtype and modality of anti-cancer therapy, two models were fit. The first included sarcoma subtypes along with binary indicators for any recent anti-cancer therapy (0–3 months versus >3 months before COVID-19 diagnosis; never or after COVID-19 diagnosis versus >3 months). The second excluded sarcoma subtypes but included binary indicators for modalities of recent anti-cancer therapy (cytotoxic chemotherapy, targeted therapy, immunotherapy, locoregional therapy—including surgery and radiation, and none). Analyses were performed using R (version 4.0.2), including the Hmisc and rms extension packages. The statistical analysis plan and data elements are provided as supplementary material. 

### 2.4. Comparison to SEER Data

Because sarcoma is an aggressive cancer, it is possible that deaths were due to the underlying cancer rather than due to COVID-19. However, capture of cause of death in CCC19 is incomplete. To isolate the impact of COVID-19 on mortality risk, we compared mortality rates among CCC19 patients with sarcoma and COVID-19 to a matched sample of patients with sarcoma reported to the Surveillance, Epidemiology, and End Results (SEER) Program database between 2000 and 2018 (and therefore before COVID-19). Exact matching was performed based on age (in decades), sex, race (Black, White, other), sarcoma subtype (bone, GIST, STS, other), cancer stage (localized, disseminated, unknown), and recency of cancer diagnosis (<1 year, 1–5 years, >5 years) using the MatchIt package in R; CCC19 patients with no match (*n* = 5) or only 1 match (*n* = 4) in SEER were excluded. For SEER patients, we defined a counterfactual time of COVID-19 diagnosis based on the median survival time for groups defined by recency of cancer diagnosis; 30 day mortality rates were calculated from this counterfactual time. Mortality at 30 days was compared between CCC19 patients and their matched SEER counterparts overall and stratified by sarcoma subtype due to expected differences in their clinical behaviors using Fisher’s exact tests.

## 3. Results

### 3.1. Clinical Characteristics of Sarcoma Patients Infected with SARS-CoV-2

From 17 March 2020 to 30 September 2021, 281 patients with sarcoma were entered into the CCC-19 registry (Figure 1) with a median follow-up of 90 days (Interquartile rage; IQR, 30–180). Baseline clinical characteristics including demographic characteristics, comorbidities, cancer-specific data, and approximate date of COVID-19 diagnosis are summarized in Table 1. Of the 281 patients, 153 (54%) were classified as having soft tissue sarcoma (STS), 48 (17%) with bone sarcoma, 45 (16%) with GIST, and 35 (13%) with other/indolent subtypes; see Supplemental Table S1 for details. The median patient age was 56 years (IQR, 41–66), 149 (53%) were male, and 133 (47%) patients were non-Hispanic white. A total of 111 (40%) patients were clinically obese and the most common comorbidity was diabetes mellitus (54 patients, 19%). A further 105 (37%) patients were reported to be in remission from their sarcoma and 152 (54%) were reported to have active sarcoma. Of patients with active sarcoma, 79 (52%) had sarcomas that were stable or responsive to current therapy, and 73 (48%) had progressing cancers. Furthermore, 53 (19%) patients had lung metastases and 37 (13%) patients had metastases to other sites. A total of 142 (51%) patients had received anticancer therapy within 3 months of their COVID-19 diagnosis, with the majority (*n* = 82, 58%) of these patients receiving cytotoxic chemotherapy. Low absolute lymphocyte count and abnormal D-Dimer levels were the most common irregular laboratory result among hospitalized patients (Supplemental Table S2).

### 3.2. Clinical Outcomes Stratified by Sarcoma Subgroup

Of the 281 patients analyzed in this study, 49% (*n* = 139) were hospitalized, 33% (*n* = 93) were hospitalized and received supplemental oxygen, 11% (*n* = 31) were admitted to the ICU, and 6% (*n* = 16) received mechanical ventilation. There were 23 deaths (8%) within 30 days of COVID-19 diagnosis and 44 (16%) deaths overall at the time of analysis. When patients were stratified by sarcoma subtype, there was only slight variation in hospitalization rates, with STS having the highest hospitalization rate at 52% (*n* = 79/153) and GIST having the lowest hospitalization rate at 44% (*n* = 20/45) (Table 2). There was no clear difference in ICU admission, supplemented oxygen, or mechanical ventilation. However, there was a small but noticeable difference in the overall death rate. Patients with GIST or other/indolent subtypes had lower rates of death (9% and 3%, respectively) when compared to bone and STS (19% and 20%, respectively).

### 3.3. Clinical Risk Factors for COVID-19 Severity

All sarcoma subtypes had lower COVID-19 severity compared to STS after adjustment (Table 3): bone sarcoma OR was 0.68 (95% CI 0.29–1.57), GIST OR was 0.37 (95% CI 0.16–0.82) and other/indolent histologies OR was 0.34 (95% CI 0.14–0.80). Timing of recent anti-cancer therapy was not associated with COVID-19 severity; recent receipt of cytotoxic chemotherapy was associated with a higher COVID-19 severity with on OR 1.97 (95% CI 0.99–3.93). Age was associated with higher COVID-19 severity (OR 1.50 per decade, 95% CI 1.17–1.93 when controlling for sarcoma subtype; OR 1.40 per decade, 95% CI 1.11–1.83 when controlling for anti-cancer modalities). Significantly higher COVID-19 severity was also observed among patients with renal disease, patients with poor ECOG performance status (2 or higher), and patients with metastases to the lung or other sites (Table 3).

### 3.4. Comparison to Matched SEER Cohort

The overall 30-day mortality rate for the CCC19 sarcoma cohort was 7.8% (20 deaths) in the CCC19 sarcoma cohort and 7.4% (1223 deaths) in the SEER sarcoma cohort. When separated by sarcoma subtype, patients with bone sarcoma and COVID-19 appeared to have a higher mortality rate than counterparts in the SEER cohort (13.5% vs 4.4%, *p* = 0.030). The 30 day mortality rates for patients with GIST, STS, or other/indolent subtypes were similar (Table 4).

## 4. Discussion

We report that patients with sarcoma have an overall high risk of developing severe COVID-19 and a higher risk of death from COVID-19 than is reported in the general population [16]. Patients with STS had higher COVID-19 severity than patients with GIST and more classically indolent histologies. Advanced age, baseline renal disease, poor performance status, and metastatic disease and site were associated with higher rates of COVID-19 complications in our multivariable analysis. Recent (within 3 months) receipt of cancer therapy was associated with high risk of COVID-19 complications, although this was not statistically significant likely due to lack of power with lower bound of the 95% confidence interval 0.99. When separated by sarcoma subtype, patients with STS and bone sarcomas had the highest likelihood of developing severe COVID-19.

One possible explanation for this difference by subtype is that STS and bone sarcomas are more likely to be treated with highly cytotoxic chemotherapy even in the curative setting, leaving some of these patients in a more immunosuppressed state over the longer term. This may be especially true for patients with bone sarcomas, who in our comparison to a matched SEER cohort had the highest excess risk of death from COVID-19. A hypothesis for this excess mortality in patients with bone sarcoma and COVID-19 is that bone sarcomas such as osteosarcoma and Ewing sarcoma are treated with prolonged, highly immunosuppressive chemotherapy that can leave the bone marrow reserve depleted in patients even after completion of therapy [17]. Our results support further investigation to test the hypothesis that depth of prior immunosuppression associates with increased risk from COVID-19. For patients with lung metastasis at presentation, surgery to resect all sites of lung metastasis after completion of chemotherapy is routinely performed, perhaps predisposing these patients to pulmonary complications of COVID-19; prior lung surgery was not routinely captured in the CCC19 registry [18].

As society begins to open without restrictions, patients with sarcoma must take this increased risk into account. COVID-19 vaccines have been shown to be effective at preventing hospitalization and death from COVID-19 [19,20,21,22]. Although some patients with cancer may have less effective immune responses to vaccination, any afforded protection may be important and a majority of those with solid tumors appear to derive benefit [23]. Among patients with sarcoma, those with STS and bone sarcomas appear to be at higher risk than other subtypes that are not typically treated with intensive chemotherapy. A potential confounding factor is that some of the patients in our cohort might have died from progressing cancer or other sequelae of sarcoma. However, a mortality rate of 8% in 30 days exceeds what would be expected from sarcoma alone. We attempted to account for this by comparing our cohort to a matched cohort from the SEER database.

Patient race has also been observed as a risk factor for adverse COVID-19 outcomes [24], including among patients with cancer [25]. Only 47% of our cohort was non-Hispanic white, suggesting that we had high minority representation. This may introduce additional confounding in interpreting our observed high COVID-19 severity rate.

Strategies to mitigate the risks faced by patients with sarcoma will be critical as cancer care delivery continues to evolve [26]. The adoption of telemedicine has the potential to decrease potential exposure to COVID-19 during peaks in cases as a significant number of cancer visits can likely be performed virtually [27]. Other strategies like pre-exposure prophylaxis, and continuing to encourage vaccination and masking in patients, relatives, and caregivers in spite of the removal of mandates can also minimize the risks faced by patients seeking cancer care.

Strengths of this study include the multi-institutional nature of the CCC19 database, inclusion of patients across a range of cancer treatment settings, and incorporation of variables not routinely available in structured electronic medical record data, such as sarcoma subtype, cancer status, and ECOG performance status. Although small compared to other CCC19 analyses, this represents a large dataset for a sarcoma-specific analysis. By comparing the survival of patients in our database to a matched cohort from SEER patients who did not have COVID-19, this is the first analysis of COVID-19 outcomes in patients with cancer to differentiate the mortality attributed to COVID-19 from the expected mortality from the cancer itself. This is important considering the overall poor long-term outcomes for patients with metastatic sarcoma.

Limitations to this analysis include its retrospective nature, selection and unmeasured confounding biases, and the rapidly changing pace of strategies for COVID-19 prevention, treatment, and management. We were not able to collect outcome data from a similar population of patients with COVID-19 who did not have cancer. A small number of those hospitalized with supplemental oxygen were known to be on chronic supplemental oxygen prior to hospitalization, such that these patients would be automatically assigned to the higher levels of ordinal severity. While this could have some type of unintended analytic effect, it is also the case that patients on chronic supplemental oxygen have pulmonary compromise and would therefore be anticipated to have more severe COVID-19 outcomes. Laboratory measurements are only somewhat informative, as most labs are not routinely checked for patients with mild COVID-19, and some labs (e.g., LDH) are even less frequently measured, with missingness >50%. Most of the patients in our cohort are from the pre-vaccination era, and it is possible that that patients at participating sites who were positive for COVID-19 were not captured in the CCC19 database. The data range of the analysis also exclude the most recent waves of cases due to the omicron variant. Future directions include assessing the impact of vaccinations and vaccine boosters, COVID-19 treatments, and practice changes made as a result of the pandemic.

## 5. Conclusions

Patients with sarcoma have high rates of severe COVID-19. Older age, poor performance status, recent cytotoxic chemotherapy, and lung metastases all contributed to worse outcomes. Patient with STS and bone sarcoma are most at risk, but only patients with bone sarcoma and COVID-19 had a higher mortality rate than patients from a matched SEER cohort. Strategies to mitigate the risk from COVID-19 are encouraged.

## Figures and Tables

**Table 1:** Patient demographic and clinical characteristics.

Characteristics	All Patients	STS	Bone	GIST	Other/Indolent
	N = 281	N = 153	N = 48	N = 45	N = 35
Age, years [Median (IQR)]	56 (41–66)	56 (43–64)	36 (22–56)	65 (60–72)	56 (38–68)
Sex					
Female	132 (47%)	80 (52%)	20 (42%)	23 (51%)	9 (26%)
Male	149 (53%)	73 (48%)	28 (58%)	22 (49%)	26 (74%)
Race/ethnicity					
Non-Hispanic White	133 (47%)	73 (48%)	24 (50%)	25 (56%)	11 (31%)
Other ^1^	145 (52%)	77 (50%)	24 (50%)	20 (44%)	24 (69%)
Missing/Unknown	3 (1%)	3 (2%)	0 (0%)	0 (0%)	0 (0%)
Obesity					
Not Obese	167 (59%)	81 (53%)	29 (60%)	30 (67%)	27 (77%)
Obese	111 (40%)	71 (46%)	18 (38%)	14 (31%)	8 (23%)
Missing/Unknown	3 (1%)	1 (1%)	1 (2%)	1 (2%)	0 (0%)
Comorbidities					
Cardiovascular	51 (18%)	29 (19%)	9 (19%)	10 (22%)	3 (9%)
Pulmonary	40 (14%)	19 (12%)	7 (15%)	11 (24%)	3 (9%)
Renal disease	14 (5%)	7 (5%)	1 (2%)	3 (7%)	3 (9%)
Diabetes Mellitus	54 (19%)	27 (18%)	11 (23%)	9 (20%)	7 (20%)
Missing/Unknown	4 (1%)	2 (1%)	1 (2%)	1 (2%)	0 (0%)
ECOG performance status					
0	78 (28%)	50 (33%)	7 (15%)	15 (33%)	6 (17%)
1	86 (31%)	53 (35%)	9 (19%)	10 (22%)	14 (40%)
2+	42 (15%)	21 (14%)	9 (19%)	7 (16%)	5 (14%)
Unknown	75 (27%)	29 (19%)	23 (48%)	13 (29%)	10 (29%)
Cancer Status					
Remission/NED	105 (37%)	45 (29%)	18 (38%)	23 (51%)	19 (54%)
Active, stable/responding	79 (28%)	42 (27%)	12 (25%)	14 (31%)	11 (31%)
Active, progressing	73 (26%)	50 (33%)	14 (29%)	7 (16%)	2 (6%)
Unknown	24 (9%)	16 (10%)	4 (8%)	1 (2%)	3 (9%)
Metastasis and lung status					
No metastatic cancer	172 (61%)	85 (56%)	25 (52%)	33 (73%)	29 (83%)
Metastatic cancer to the lung	53 (19%)	38 (25%)	12 (25%)	3 (7%)	0 (0%)
Metastatic cancer—other sites	37 (13%)	19 (12%)	8 (17%)	5 (11%)	5 (14%)
Missing/Unknown	19 (7%)	11 (7%)	3 (6%)	4 (9%)	1 (3%)
Modality of Recent Anti-cancer Therapy (within 3 months prior to COVID-19 diagnosis)					
None within 3 months of COVID-19	137 (49%)	72 (47%)	21 (44%)	19 (42%)	25 (71%)
Cytotoxic Therapy	82 (29%)	53 (35%)	22 (46%)	3 (7%)	4 (11%)
Anthracycline ^2^	16 (6%)	9 (6%)	4 (8%)	1 (2%)	2 (6%)
Targeted Therapy	44 (16%)	13 (8%)	6 (12%)	22 (49%)	3 (9%)
TKI	31 (11%)	10 (7%)	4 (8%)	17 (38%)	0 (0%)
VEGF Inhibitor	13 (5%)	8 (5%)	2 (4%)	3 (7%)	0 (0%)
Endocrine Therapy	4 (1%)	2 (1%)	1 (2%)	1 (2%)	0 (0%)
Immunotherapy	10 (4%)	8 (5%)	1 (2%)	1 (2%)	0 (0%)
Local	41 (15%)	27 (18%)	7 (15%)	4 (9%)	3 (9%)
Other	2 (1%)	0 (0%)	1 (2%)	1 (2%)	0 (0%)
Missing/Unknown	2 (1%)	1 (1%)	1 (2%)	0 (0%)	0 (0%)
Timing of anti-cancer therapy					
Never or after COVID-19 diagnosis	24 (9%)	14 (9%)	6 (12%)	3 (7%)	1 (3%)
0–4 weeks before COVID-19 diagnosis	113 (40%)	60 (39%)	23 (48%)	21 (47%)	9 (26%)
1–3 months before COVID-19 diagnosis	29 (10%)	20 (13%)	3 (6%)	5 (11%)	1 (3%)
More than 3 months before COVID-19 diagnosis	106 (38%)	52 (34%)	15 (31%)	16 (36%)	23 (66%)
Missing/Unknown	9 (3%)	7 (5%)	1 (2%)	0 (0%)	1 (3%)
Date of COVID-19 Diagnosis					
January–April 2020	44 (16%)	21 (14%)	5 (10%)	9 (20%)	9 (26%)
May–August 2020	125 (44%)	72 (47%)	28 (58%)	16 (36%)	9 (26%)
September–December 2020	65 (23%)	37 (24%)	8 (17%)	9 (20%)	11 (31%)
January–April 2021	43 (15%)	22 (14%)	7 (15%)	8 (18%)	6 (17%)
May–September 2021	4 (1%)	1 (1%)	0 (0%)	3 (6%)	0 (0%)

^1^ All other than Non-Hispanic White includes American Indian/Alaska Native, Asian, Native Hawaiian or Other Pacific Islander, Black or African American, Hispanic. ^2^ Patients receiving anthracyclines were included in the total count of patients receiving cytotoxic therapy.

**Table 2:** Outcomes for patients with sarcoma and COVID-19.

Outcomes	All Patients	STS	Bone	GIST	Other/Indolent
	N = 281	N = 153	N = 48	N = 45	N = 35
Hospitalization	139 (49%)	79 (52%)	24 (50%)	20 (44%)	16 (46%)
Without supplemental O_2_	43 (15%)	27 (18%)	7 (15%)	3 (7%)	6 (17%)
With Supplemental O_2_^1^	93 (33%)	50 (33%)	16 (33%)	17 (38%)	10 (29%)
Intensive care unit admission	31 (11%)	19 (12%)	4 (8%)	5 (11%)	3 (9%)
Received mechanical ventilation	16 (6%)	8 (5%)	4 (8%)	3 (7%)	1 (3%)
Death due to any cause					
Within 30 days	23 (8%)	13 (8%)	7 (15%)	2 (4%)	1 (3%)
Ever during follow-up	44 (16%)	30 (20%)	9 (19%)	4 (9%)	1 (3%)
Follow up time, days [Median (IQR)]	90 (30–180)	90 (30–180)	63 (29–180)	90 (30–180)	180 (36–225)

^1^ Seven patients assigned to the hospitalized with oxygen ordinal severity had a baseline oxygen requirement, and three had an unknown baseline oxygen requirement.

**Table 3:** Analysis of clinical predictors for patients with sarcoma and COVID19.

	With Sarcoma Subtypes		With Anti-Cancer Modalities	
	*Odds Ratios*	*95% CI*	*Odds Ratios*	*95% CI*
Sarcoma subtype				
Bone vs. STS	0.68	0.29–1.57		
GIST vs. STS	0.37	0.16–0.82		
Other/Indolent histologies vs STS	0.34	0.14–0.83		
Timing of any anti-cancer therapy				
0–3 months vs. >3 months before COVID-19 diagnosis	1.59	0.81–3.14		
Never or after COVID diagnosis vs. >3 months before COVID-19 diagnosis	1.08	0.38–3.08		
Modality of recent anti-cancer therapy				
Cytotoxic therapy (yes vs. no)			1.97	0.99–3.93
Targeted therapy (yes vs. no)			0.97	0.46–2.03
Immunotherapy (yes vs. no)			2.03	0.46–8.99
Locoregional therapy (yes vs. no)			1.56	0.73–3.32
No therapy, ever vs. >3 months before COVID-19 diagnosis			1.34	0.50–3.59
Age, per decade				
<40 years	0.87	0.48–1.59	0.97	0.54–1.74
>40 years	1.50	1.17–1.93	1.43	1.11–1.83
Sex (Male vs. Female)	1.71	0.98–2.99	1.51	0.87–2.60
Renal disease (yes vs. no)	3.69	1.25–10.9	2.92	0.94–9.03
ECOG performance status				
1 vs. 0	2.01	0.97–4.16	1.74	0.85-3.58
2+ vs. 0	20.7	8.24–52.0	17.4	7.01–43.2
Unknown vs. 0	2.98	1.35–6.54	2.60	1.22–5.55
Metastasis				
To lung vs. none	5.69	2.46–13.2	6.47	2.80–15.0
To other sites vs. none	2.94	1.29–6.70	2.80	1.19–6.60
Date of COVID-19 Diagnosis				
May–August 2020 vs. January–April 2020	0.41	0.19–0.90	0.41	0.19–0.89
September–December 2020 vs. January–April 2020	0.14	0.06–0.33	0.14	0.06–0.33
January–September 2021 vs. January–April 2020	0.31	0.12–0.78	0.27	0.11–0.69

**Table 4:** 30 day Mortality Rates for CCC19 and matched SEER Sarcoma Patients, Overall and by Subtype.

30 Day Mortality	CCC19			SEER			
	N Cases	*n* Deaths	Mortality Rate (%)	N Cases	*n* Deaths	Mortality Rate (%)	*p*-Value ^1^
Overall	255	20	7.8%	16582	1223	7.4%	0.718
Sarcoma Subtype							
Bone	37	5	13.5%	608	27	4.4%	0.030
GIST	43	2	4.7%	1408	52	3.7%	0.673
STS	144	12	8.3%	13,689	1123	8.2%	0.879
Other	31	1	3.2%	877	21	2.4%	0.539

^1^ Fisher’s exact test.

## Data Availability

Restrictions apply to the availability of these data. Data were obtained from CCC19 and are available from the authors with the permission of the CCC19 Steering Committee.

## Supplementary Materials

The following supporting information can be downloaded at: https://www.mdpi.com/article/10.3390/cancers14174334/s1, Table S1: Sarcoma subtype at diagnosis and classification into sarcoma subgroups; Table S2: Laboratory results among hospitalized patients. File S1: List of participants by institution: Alphabetical list of participants by institution that contributed at least one record to the analysis.
