# Supplementary material for: Demographics, Outcomes, and Risk Factors for Patients with Sarcoma and COVID-19: A CCC19-Registry Based Retrospective Cohort Study
Source: Cancers (Basel). 2022 Sep 5;14(17):4334. doi: 10.3390/cancers14174334 (PMC9454925; doi:10.3390/cancers14174334)
Supplement: Supplementary file 1 [file cancers-14-04334-s001.zip › Supplemental File S1.pdf]

Appendix A

List of participants by institution: Alphabetical list of participants by institution that contributed at least one record to the analysis.

Bolded = site PI/co-PIs; site co-investigators are listed alphabetically by last name.

- **Balazs Halmos, MD; Amit Verma, MBBS**; Benjamin A. Gartrell, MD; Sanjay Goel, MBBS; Nitin Ohri, MD; R. Alejandro Sica, MD; Astha Thakkar, MD (Albert Einstein College of Medicine, Montefiore Medical Center, Bronx, NY, USA)
- **Sigrun Hallmeyer, MD**; Pamela Bohachek, RN, CCRC; Daniel Mundt, MD; Sasirekha Pandravada, DO; Mauli Patel, DO; Mitrianna Streckfuss, MPH; Eyob Tadesse, MD; Michael A. Thompson, MD, PhD, FASCO (Aurora Cancer Care, Advocate Aurora Health, Milwaukee, WI, USA)
- **Jonathan M. Loree, MD, MS, FRCPC**; Irene S. Yu, MD, FRCPC (BC Cancer, Vancouver, BC, Canada)
- **Mary Linton B. Peters, MD, MS, FACP**; Poorva Bindal, MD; Andrew J. Piper-Vallillo, MD (Beth Israel Deaconess Medical Center, Boston, MA, USA)
- **Dimitrios Farmakiotis, MD, FACP, FIDSA**; Panos Arvanitis, MS; Pamela C. Egan, MD; Hina Khan, MD; Elizabeth J. Klein, BA; Adam J. Olszewski, MD; Kendra Vieira, BS (Brown University and Lifespan Cancer Institute, Providence, RI, USA)
- **Salvatore A. Del Prete, MD**; Michael H. Bar, MD, FACP; Anthony P. Gulati, MD; K. M. Steve Lo, MD; Suzanne J. Rose, MS, PhD, CCRC, FACRP; Jamie Stratton, MD; Paul L. Weinstein, MD (Carl & Dorothy Bennett Cancer Center at Stamford Hospital, Stamford, CT, USA)
- **Shilpa Gupta, MD**; Nathan A. Pennell, MD, PhD, FASCO; Manmeet S. Ahluwalia, MD, FACP; Scott J. Dawsey, MD; Christopher A. Lemmon, MD; Amanda Nizam, MD; Nima Sharifi, MD (Cleveland Clinic, Cleveland, OH, USA)
- **Toni K. Choueiri, MD**; Ziad Bakouny, MD, MSc; Jean M. Connors, MD; George D. Demetri, MD, FASCO; Dory A. Freeman, BS; Antonio Giordano, MD, PhD; Chris Labaki, MD; Alicia K. Morgans, MD, MPH; Anju Nohria, MD; Renee-Maria Saliby, MD, MSc; Andrew L. Schmidt, MD; Eliezer M. Van Allen, MD; Wenxin (Vincent) Xu, MD; Rebecca L. Zon, MD (Dana-Farber Cancer Institute, Boston, MA, USA)
- **Gary H. Lyman, MD, MPH, FASCO, FRCP**; Jerome J. Graber MD, MPH; Petros Grivas, MD, PhD; Jessica E. Hawley, MD; Elizabeth T. Loggers, MD, PhD; Ryan C. Lynch, MD; Elizabeth S. Nakasone, MD, PhD; Michael T. Schweizer, MD; Lisa Tachiki, MD; Shaveta Vinayak, MD, MS; Michael J. Wagner, MD; Albert Yeh, MD (Fred Hutchinson Cancer Research Center/University of Washington/Seattle Cancer Care Alliance, Seattle, WA, USA)
- **Na Tosha N. Gatson, MD, PhD, FAAN**; Yvonne Dansoa, DO; Mina Makary, MD; Jesse J. Manikowski, MS; Joseph Vadakara, MD; Kristena Yossef, MD (Geisinger Health System, PA, USA)
- **Sharad Goyal, MD**; Minh-Phuong Huynh-Le, MD, MAS (George Washington University, Washington, DC, USA)
- **Lori J. Rosenstein, MD** (Gundersen Health System, WI, USA)
- **Peter Paul Yu, MD, FACP, FASCO**; Jessica M. Clement, MD; Ahmad Daher, MD; Mark E. Dailey, MD; Rawad Elias, MD; Asha Jayaraj, MD; Emily Hsu, MD; Alvaro G. Menendez, MD; Oscar K. Serrano, MD, MBA, FACS (Hartford HealthCare Cancer Institute, Hartford, CT, USA)
- **Clara Hwang, MD**; Shirish M. Gadgeel, MD; Sheela Tejwani, MD (Henry Ford Cancer Institute, Henry Ford Hospital, Detroit, MI, USA)
- **Melissa K. Accordino, MD, MS**; Divaya Bhutani, MD; Dawn Hershman, MD, MS, FASCO; Matthew A. Ingham, MD; Gary K. Schwartz, MD (Herbert Irving Comprehensive Cancer Center at Columbia University, New York, NY, USA)

- **Daniel Y. Reuben, MD, MS**; Mariam Alexander, MD, PhD; Sara Matar, MD; Sarah Mushtaq, MD (Hollings Cancer Center at the Medical University of South Carolina, Charleston, SC, USA)
- **Eric H. Bernicker, MD** (Houston Methodist Cancer Center, Houston, TX, USA)
- **Erika Ruíz-García, MD, MCs**; Ana Ramirez, MD; Diana Vilar-Compte, MD, MsC (Instituto Nacional de Cancerologia, Mexico City, Mexico)
- **Mark A. Lewis, MD; Terence D. Rhodes, MD, PhD**; David M. Gill, MD; Clarke A. Low, MD (Intermountain Health Care, Salt Lake City, UT, USA)
- **Sandeep H. Mashru, MD**; Abdul-Hai Mansoor, MD (Kaiser Permanente Northwest, OR/WA, USA)
- **Brandon Hayes-Lattin, MD, FACP**; Aaron M. Cohen, MD, MS; Shannon McWeeney, PhD; Eneida R. Nemecek, MD, MS, MBA; Staci P. Williamson, BS (Knight Cancer Institute at Oregon Health and Science University, Portland, OR, USA)
- **Gayathri Nagaraj, MD**; Mojtaba Akhtari, MD; Dan R. Castillo, MD; Eric Lau, DO; Mark E. Reeves, MD, PhD (Loma Linda University Cancer Center, Loma Linda, CA, USA)
- **Stephanie Berg, DO**; Natalie Knox, BS; Timothy E. O'Connor, MD (Loyola University Medical Center, Maywood, IL, USA)
- **Firas H. Wehbe, MD, PhD**; Jessica Altman, MD; Michael Gurley, BA; Mary F. Mulcahy, MD (Lurie Cancer Center at Northwestern University, Chicago, IL, USA)
- **Eric B. Durbin, DrPH, MS** (Markey Cancer Center at the University of Kentucky, Lexington, KY, USA)
- **Amit A. Kulkarni, MD**; Heather H. Nelson, PhD, MPH; Zohar Sachs, MD, PhD (Masonic Cancer Center at the University of Minnesota, Minneapolis, MN, USA)
- **Thorvardur R. Halfdanarson, MD**; Tanios S. Bekaii-Saab, MD, FACP; Aakash Desai, MD, MPH; Irbaz B. Riaz, MD, MS; Surbhi Shah, MD; Katherine E. Smith, MD; Colt Williams, MD; Zhuoer Xie, MD, MS (Mayo Clinic, AZ/FL/MN, USA)
- **Ruben A. Mesa, MD, FACP**; Mark Bonnen, MD; Daruka Mahadevan, MD, PhD; Amelie G. Ramirez, DrPH, MPH; Mary Salazar, DNP, MSN, RN, ANP-BC; Dimpy P. Shah, MD, PhD; Pankil K. Shah, MD, MSPH (Mays Cancer Center at UT Health San Antonio MD Anderson Cancer Center, San Antonio, TX, USA)
- **Gregory J. Riely, MD, PhD**; **Elizabeth V. Robilotti MD, MPH**; Rimma Belenkaya, MA, MS; John Philip, MS (Memorial Sloan Kettering Cancer Center, New York, NY, USA)
- **Bryan Faller, MD** (Missouri Baptist Medical Center, St. Louis, MO, USA)
- **Rana R. McKay, MD**; Archana Ajmera, MSN, ANP-BC, AOCNP; Sharon S. Brouha, MD, MPH; Angelo Cabal, BS; Sharon Choi, MD, PhD; Albert Hsiao, MD, PhD; Jun Yang Jiang, MD; Seth Kligerman, MD; Taylor K. Nonato; Erin G. Reid, MD (Moores Comprehensive Cancer Center at the University of California, San Diego, La Jolla, CA, USA)
- **Lisa B. Weissmann, MD**; Padmanabh S. Bhatt, MD; Chinmay Jani, MD; Melissa G. Mariano, DO; Carey C. Thomson, MD, FCCP, MPH (Mount Auburn Hospital, Cambridge, MA, USA)
- **Sachin R. Jhawar, MD**; Daniel Addison, MD; James L. Chen, MD; Margaret E. Gatti-Mays, MD; Vidhya Karivedu, MBBS; Joshua D. Palmer, MD; Daniel G. Stover, MD; Sarah Wall, MD; Nicole O. Williams, MD (The Ohio State University Comprehensive Cancer Center, Columbus, OH, USA)
- **Monika Joshi, MD, MRCP**; Hyma V. Polimera, MD; Lauren D. Pomerantz; Marc A. Rovito, MD, FACP (Penn State Health/Penn State Cancer Institute/St. Joseph Cancer Center, PA, USA)
- **Elizabeth A. Griffiths, MD**; Pragati G. Advani, MD, MPH; Igor Puzanov, MD, MSCI, FACP (Roswell Park Comprehensive Cancer Center, Buffalo, NY, USA)
- **Gerald Batist, MD, FACP, FRCP**; Erin Cook, MSN; Miriam Santos Dutra, PhD; Cristiano Ferrario, MD; Wilson H. Miller Jr., MD, PhD (Segal Cancer Centre, Jewish General Hospital, McGill University, Montreal, QC, Canada)

- **Babar Bashir, MD, MS**; Christopher McNair, PhD; Sana Z. Mahmood, BA, BS; Vasil Mico, BS; Andrea Verghese Rivera, MD (Sidney Kimmel Cancer Center at Thomas Jefferson University, Philadelphia, PA, USA)
- **Sumit A. Shah, MD, MPH**; Elwyn C. Cabebe, MD; Michael J. Glover, MD; Alokkumar Jha, PhD; Ali Raza Khaki, MD; Lidia Schapira, MD, FASCO; Julie Tsu-Yu Wu, MD, PhD (Stanford Cancer Institute at Stanford University, Palo Alto, CA, USA)
- **Suki Subbiah, MD** (Stanley S. Scott Cancer Center at LSU Health Sciences Center, New Orleans, LA, USA)
- **Daniel B. Flora, MD, PharmD**; Goetz Kloecker, MD; Barbara B. Logan, MS; Chaitanya Mandapakala, MD (St. Elizabeth Healthcare, Edgewood, KY, USA)
- **Natasha C. Edwin, MD**; Melissa Smits, APC (ThedaCare Cancer Care, Appleton, WI, USA)
- **Alyson Fazio, APRN-BC**; Julie C. Fu, MD; Kathryn E. Huber, MD; Mark H. Sueyoshi, MD (Tufts Medical Center Cancer Center, Boston and Stoneham, MA, USA)
- **Vadim S. Koshkin, MD**; Hala T. Borno, MD; Daniel H. Kwon, MD; Eric J. Small, MD; Sylvia Zhang, MS (UCSF Helen Diller Family Comprehensive Cancer Center at the University of California at San Francisco, CA, USA)
- **Samuel M. Rubinstein, MD**; **William A. Wood, MD, MPH**; Tessa M. Andermann, MD; Christopher Jensen, MD (UNC Lineberger Comprehensive Cancer Center, Chapel Hill, NC, USA)
- **Trisha M. Wise-Draper, MD, PhD**; Syed A. Ahmad, MD, FACS; Punita Grover, MD; Shuchi Gulati, MD; Jordan Kharofa, MD; Tahir Latif, MBBS, MBA; Michelle Marcum, MS; Hira G. Shaikh; MD; Davendra P. S. Sohal, MD, MPH; Olga Zamulko, MD (University of Cincinnati Cancer Center, Cincinnati, OH, USA)
- **Daniel W. Bowles, MD**; Christoper L. Geiger, MD (University of Colorado Cancer Center, Aurora, CO, USA)
- **Merry-Jennifer Markham, MD, FACP, FASCO**; Atlantis D. Russ, MD, PhD; Haneen Saker, MD (University of Florida Health Cancer Center, Gainesville, FL, USA)
- **Lawrence E. Feldman, MD; Kent F. Hoskins, MD**; Gerald Gantt Jr., MD; Li C. Liu, PhD; Mahir Khan, MD; Ryan H. Nguyen, DO; Mary Pasquinelli, APN, DNP; Candice Schwartz, MD; Neeta K. Venepalli, MD, MBA (University of Illinois Hospital & Health Sciences System, Chicago, IL, USA)
- **Elizabeth Wulff-Burchfield, MD**; Anup Kasi MD, MPH; Crosby D. Rock, MD (The University of Kansas Cancer Center, Kansas City, KS, USA)
- **Christopher R. Friese, PhD, RN, AOCN, FAAN; Leslie A. Fecher, MD** (University of Michigan Rogel Cancer Center, Ann Arbor, MI, USA)
- **Sonya A. Reid, MD, MPH**; Alicia Beeghly, MPH, PhD; Alaina J. Brown, MD, MPH; Alex Cheng, PhD; Sarah Croessmann, PhD; Elizabeth J. Davis, MD; Kyle T. Enriquez, MSc BS; Erin A. Gillaspie, MD, MPH; Daniel Hausrath, MD; Douglas B. Johnson, MD, MSCI; Xuanyi Li, MD; Sanjay Mishra, MS, PhD; Brian I. Rini, MD, FACP, FASCO; David A. Slosky, MD; Carmen C. Solorzano, MD, FACS; Matthew D. Tucker, MD; Karen Vega-Luna, MA; Lucy L. Wang, BA (Vanderbilt-Ingram Cancer Center at Vanderbilt University Medical Center, Nashville, TN, USA)
- **Matthew Puc, MD**; Theresa M. Carducci, MSN, RN, CCRP; Karen J. Goldsmith, BSN, RN; Susan Van Loon, RN, CTR, CCRP (Virtua Health, Marlton, NJ, USA)
- **Prakash Peddi, MD; Lane R. Rosen, MD**; Briana Barrow McCollough, BSc, CCRC (Willis-Knighton Cancer Center, Shreveport, LA, USA)
- **Mehmet A. Bilen, MD**; Cecilia A. Castellano; Deepak Ravindranathan, MD, MS (Winship Cancer Institute of Emory University, Atlanta, GA, USA)
