# Supplementary material for: Demographics, Outcomes, and Risk Factors for Patients with Sarcoma and COVID-19: A CCC19-Registry Based Retrospective Cohort Study
Source: Cancers (Basel). 2022 Sep 5;14(17):4334. doi: 10.3390/cancers14174334 (PMC9454925; doi:10.3390/cancers14174334)
Supplement: Supplementary file 1 [file cancers-14-04334-s001.zip › Supplemental Tables S1 and S2.pdf]

Supplemental Table S1: Sarcoma subtype at diagnosis and classification into sarcoma subgroups.

| Sarcoma Type | All subjects N = 281 | STS N = 153 | Bone N = 48 | GIST N = 45 | Other/Indolent N = 35 |
|---|---|---|---|---|---|
| Angiosarcoma | 5 (2%) | 5 (3%) | 0 (0%) | 0 (0%) | 0 (0%) |
| Bone cancer, NOS | 8 (3%) | 0 (0%) | 8 (17%) | 0 (0%) | 0 (0%) |
| Dedifferentiated liposarcoma | 14 (5%) | 14 (9%) | 0 (0%) | 0 (0%) | 0 (0%) |
| Ewing sarcoma | 14 (5%) | 0 (0%) | 14 (29%) | 0 (0%) | 0 (0%) |
| GIST | 45 (16%) | 0 (0%) | 0 (0%) | 45 (100%) | 0 (0%) |
| Kaposi sarcoma | 18 (6%) | 0 (0%) | 0 (0%) | 0 (0%) | 18 (51%) |
| Malignant peripheral nerve sheath tumor (MPNST) | 5 (2%) | 5 (3%) | 0 (0%) | 0 (0%) | 0 (0%) |
| Multiple | 5 (2%) | 5 (3%) | 0 (0%) | 0 (0%) | 0 (0%) |
| Myxoid/round cell liposarcoma | 7 (2%) | 7 (5%) | 0 (0%) | 0 (0%) | 0 (0%) |
| Non-uterine Leiomyosarcoma | 16 (6%) | 16 (10%) | 0 (0%) | 0 (0%) | 0 (0%) |
| Osteosarcoma | 26 (9%) | 0 (0%) | 26 (54%) | 0 (0%) | 0 (0%) |
| Rhabdomyosarcoma | 3 (1%) | 3 (2%) | 0 (0%) | 0 (0%) | 0 (0%) |
| Soft tissue sarcoma NOS | 12 (4%) | 12 (8%) | 0 (0%) | 0 (0%) | 0 (0%) |
| Spindle cell/soft tissue sarcoma NOS | 16 (6%) | 16 (10%) | 0 (0%) | 0 (0%) | 0 (0%) |
| STS or Vascular - Other | 27 (10%) | 27 (18%) | 0 (0%) | 0 (0%) | 0 (0%) |
| STS or Vascular - Unknown | 1 (0%) | 1 (1%) | 0 (0%) | 0 (0%) | 0 (0%) |
| Synovial sarcoma | 11 (4%) | 11 (7%) | 0 (0%) | 0 (0%) | 0 (0%) |
| Undifferentiated pleomorphic sarcoma (UPS)/ Myxofibrosarcoma | 25 (9%) | 25 (16%) | 0 (0%) | 0 (0%) | 0 (0%) |
| Uterine leiomyosarcoma | 6 (2%) | 6 (4%) | 0 (0%) | 0 (0%) | 0 (0%) |
| Well differentiated liposarcoma | 16 (6%) | 0 (0%) | 0 (0%) | 0 (0%) | 16 (46%) |
| Missing/Unknown | 1 (0%) | 0 (0%) | 0 (0%) | 0 (0%) | 1 (3%) |

Supplemental Table S2: Laboratory results among hospitalized patients.

| Characteristics | All hospitalized subjects N = 122 | STS N = 66 | Bone N = 21 | GIST N = 19 | Other/Indolent N = 16 |
|---|---|---|---|---|---|
| Absolute lymphocyte count | | | | | |
| Low | 50 (41%) | 29 (44%) | 8 (38%) | 7 (37%) | 6 (38%) |
| Normal | 39 (32%) | 18 (27%) | 9 (43%) | 10 (53%) | 2 (12%) |
| High | 0 (0%) | 0 (0%) | 0 (0%) | 0 (0%) | 0 (0%) |
| Missing/Unknown | 33 (27%) | 19 (29%) | 4 (19%) | 2 (11%) | 8 (50%) |
| Absolute neutrophil count | | | | | |
| Low | 6 (5%) | 4 (6%) | 1 (5%) | 1 (5%) | 0 (0%) |
| Normal | 63 (52%) | 34 (52%) | 10 (48%) | 11 (58%) | 8 (50%) |
| High | 21 (17%) | 10 (15%) | 6 (29%) | 5 (26%) | 0 (0%) |
| Missing/Unknown | 32 (26%) | 18 (27%) | 4 (19%) | 2 (11%) | 8 (50%) |
| White Blood Cell Count | | | | | |
| Low | 27 (22%) | 13 (20%) | 4 (19%) | 5 (26%) | 5 (31%) |
| Normal | 57 (47%) | 33 (50%) | 11 (52%) | 8 (42%) | 5 (31%) |
| High | 16 (13%) | 9 (14%) | 3 (14%) | 4 (21%) | 0 (0%) |
| Missing/Unknown | 22 (18%) | 11 (17%) | 3 (14%) | 2 (11%) | 6 (38%) |
| Creatinine | | | | | |
| Normal | 78 (64%) | 46 (70%) | 13 (62%) | 12 (63%) | 7 (44%) |
| Abnormal | 20 (16%) | 8 (12%) | 4 (19%) | 5 (26%) | 3 (19%) |
| Missing/Unknown | 24 (20%) | 12 (18%) | 4 (19%) | 2 (11%) | 6 (38%) |
| LDH | | | | | |
| Normal | 20 (16%) | 9 (14%) | 3 (14%) | 5 (26%) | 3 (19%) |
| Abnormal | 33 (27%) | 15 (23%) | 7 (33%) | 6 (32%) | 5 (31%) |
| Missing/Unknown | 69 (57%) | 42 (64%) | 11 (52%) | 8 (42%) | 8 (50%) |
| D-Dimer | | | | | |
| Normal | 10 (8%) | 5 (8%) | 3 (14%) | 1 (5%) | 1 (6%) |
| Abnormal | 47 (39%) | 27 (41%) | 5 (24%) | 9 (47%) | 6 (38%) |
| Missing/Unknown | 65 (53%) | 34 (52%) | 13 (62%) | 9 (47%) | 9 (56%) |
